# Ophthalmic Manifestations of Leukemia in a Tertiary Hospital Population of Adult Nigerian Africans

**DOI:** 10.4103/0974-9233.71599

**Published:** 2010

**Authors:** Boniface I. Eze, Godswill O. Ibegbulam, Sunday Ocheni

**Affiliations:** Department of Ophthalmology, University of Nigeria Teaching Hospital (UNTH), Ituku-Ozalla, PMB 01139, Enugu, Nigeria; 1 Department of Hematology and Immunology, University of Nigeria Teaching Hospital (UNTH), Ituku-Ozalla, PMB 01139, Enugu, Nigeria

**Keywords:** Leukemia, Leukemic Ophthalmopathy, Nigeria, Vision Loss

## Abstract

**Purpose:**

To determine the prevalence and pattern of leukemic ophthalmopathy among adults at the University of Nigeria Teaching Hospital (UNTH), Enugu, south-eastern, Nigeria.

**Materials and Methods:**

This prospective, observational case series surveyed adult leukemia patients presenting at UNTH’s departments of Hematology/Immunology and Ophthalmology from July 2003 to August 2008. The demographic profile, clinical data from for each individual in the cohort were statistically collated and analyzed. A *P* <0.05 was considered as statistically significant.

**Results:**

There were 72 participants (45 males and 27 females), aged 32.7 ± 9.8 years (range, 18 years to 72 years). Leukemic ophthalmopathy was present in 77.8% of subjects. The leading ophthalmic manifestations of leukemia were retinal vascular abnormalities in 50.0% of subjects, conjunctival pallor in 27.8% of subjects, sub-conjunctival hemorrhage in 19.4% of subjects, and retinal hemorrhage in 16.7% of subjects. Ocular co-morbidity was present in 47.2% of subjects. Vision loss occurred in 37.5% of subjects, of which 32.1% was leukemia related, and the remaining due to ocular co-morbidity. Leukemic ophthalmopathy was more prevalent in chronic leukemia (*P* <0.05), frequently affected the ocular posterior segment (*P* < 0.05), and often resulted from secondary hematologic complications (*P* <0.05). There was no gender difference in the prevalence of leukemia (*P* = 0.0822) or leukemic ophthalmopathy (*P* = 0.6624).

**Conclusion:**

The prevalence of leukemic ophthalmopathy in Enugu is high. It is often associated with significant ocular co-morbidity and vision loss. These have implications for clinicians involved in leukemia management. Early diagnosis and regular ophthalmic examinations are recommended to optimize treatment outcomes.

## INTRODUCTION

Leukemia is a malignant proliferative disorder of leucopoietic bone marrow stem cells characterized by over-crowding of the bone marrow by immature neoplastic leucocytes and widespread infiltration of organs, tissues, and peripheral blood by immature leucocytes.^1^ The resultant displacement of normal hematopoietic stem cells from the bone marrow leads to secondary hematologic complications such as erythropenia, thrombocytopenia and leukostasis. Although there is peripheral leucocytosis, the circulating leucocytes are immature and dysfunctional. These secondary hematologic alterations are responsible for tissue ischemia, bleeding diasthesis, immune-suppression, and hyper-viscosity state which are the cardinal pathologic features of leukemia.^2^

Based on cell type, differentiation, morphology, cyto-chemical characteristics, and immune-phenotyping, leukemia is classified into acute and chronic subgroups. Each sub-group is further sub-divided into myeloblastic and myelocytic variants such as acute lymphoblastic leukemia (ALL), acute myeloblastic leukemia (AML), chronic lymphocytic leukemia (CLL) and chronic myelocytic leukemia (CML). However, a variant called bi-phenotypic leukemia (BPL) exhibits features of acute and chronic leukemia.^1^–^3^

Leukemic ophthalmopathy, symptomatic or asymptomatic, may result from direct ocular infiltration by leukemic cells, indirect ocular involvement resulting from secondary hematologic changes, opportunistic infections and complications of various modalities of therapy i.e. cytotoxic drugs, total body irradiation (TBI), and allogenic bone marrow transplantation (BMT).^4^–^8^ The ophthalmic manifestations of leukemia include ocular, orbital, adnexal, or neuro-ophthalmic manifestation, usually correlate with systemic disease during blast crisis. However, these may manifest prior to systemic disease or signal an isolated focal relapse after complete recovery from systemic leukemia.^3^,^4^,^9^ Ophthalmic involvement in leukemia is associated with significant ocular morbidity and vision loss, and has critical implications for the natural course and survival prognosis of systemic leukemia.

Although previous studies performed outside Nigeria^4^,^9^–^11^ have reported the prevalence of leukemic ophthalmopathy in the range of 9-90%, there is a relative paucity of research on this topic from Africa. In this study, we studied the prevalence and spectrum of leukemic ophthalmopathy among adults in Enugu, southeastern Nigeria. We conducted a five-year prospective ophthalmic evaluation of consecutive adult hospital-based patients with leukemia. The findings would further acquaint ophthalmologists, practicing in Africa or farther afield, with the pattern of ophthalmic manifestations of leukemia in adult black Africans and also inform local eye care providers on the optimal resource needs for adequate management.

## MATERIALS AND METHODS

The cohort comprised consecutive new and existing subjects aged 16 years and above presenting at the Hematology/Immunology clinic of the University of Nigeria Teaching Hospital (UNTH), Enugu, between July 2003 and August 2008. In all cases the diagnosis of leukemia was confirmed with a bone marrow biopsy. Hematologic diagnosis was peformed by two consultant hematologists (GOI and SO), before referral to the UNTH’s eye clinic for ophthalmic evaluation by a consultant ophthalmologist (BIE).

Demographic data, type of leukemia, duration of illness, and modality of treatment were obtained from their Hematology/Immunology clinical charts.

The ophthalmic evaluation protocol consisted of ophthalmic history, detailed ophthalmic examination including measurement of best corrected distant visual acuity (BCVA) with a retro-illuminated Snellen distance visual acuity chart with English alphabet or Tumbling E distant (Unique Opticals Inc, Lagos, Nigeria). Visual acuity was categorized as normal vision (unaided distance visual acuity of 6/6-6/18), visual impairment (BCVA < 6/18 in the better eye), and acuity blindness (BCVA < 3/60 in the better eye). Other ophthalmic exams included slit lamp biomicroscopy (Haag-Streit Inc, Berne, Switzerland) of ocular anterior segment, ocular adnexa, and anterior vitreous, gonioscopy with the Goldman 3-mirror lens (Haag-Streit Inc, Berne, Switzerland), pre-corneal tear film evaluation using the Schirmer tear secretion test using filter paper (TearFlo®, Contacare Ophthalmic Inc; Gujarat, India), tear film break up time (TFBUT) assessment with one drop in each eye of 2% sodium fluorescein (ECWA Laboratories, Jos, Nigeria), pupillary light reaction with a penlight (Keeler Ltd., Winsor, UK), applanation tonometry with the Perkins hand-held tonometer (Clement Clark International, Harlow, Essex, UK) after instillation of one drop each of 2% proparacaine (Ashford Laboratories Inc, Mumbai, India) and 2% sodium fluorescein (ECWA Laboratories Inc, Jos, Nigeria) bilaterally.

Pupillary dilatation was subsequently performed with one drop in each eye of 1% tropicamide (Mydriacyl® Alcon Inc, Fort Worth, Tx, USA) and 2.5% phenylepherine (Mydfrin® Alcon Inc, Fort Worth, Tx, USA) followed by examination of the posterior segment with a direct ophthalmoscope (Heine Optotechnik, Herrsching, Germany), slit lamp indirect ophthalmoscopy using +78D lens (Ocular Instruments Inc, Texas, USA), and binocular indirect ophthalmoscope (Keeler Ltd., Winsor, UK). Leukemia-related and non-leukemia ophthalmic findings were documented for each subject. Non-ambulatory leukemia inpatients were examined at bedside and had a further ophthalmic examination, at the eye clinic if warranted.

Data was captured based on a study protocol, entered and analyzed using the Statistical Package for Social Sciences software, version 17, (SPSS Inc, Chicago, USA) to generate frequencies, percentages, and proportions. Associations between variables were examined for statistical significance using the *Chi*- square. A *P* < 0.05 (with one degree of freedom) was considered as statistically significant.

Prior to commencement of the study, ethical clearance was received from the UNTH’s Ethical Committee. This study adhered to the 1964 Declaration of Helsinki on research on human subjects. Informed consent was obtained from potential study subjects before recruitment into the study. Exclusion criteria included refusal of subjects to participate, co-existing ocular or systemic disease with leukemia-like ocular manifestations e.g. HIV/AIDS, diabetes mellitus, systemic hypertension, sickle cell disease, and retinal vascular disease, presence of fundus-obscuring opacity in the ocular media, and severely ill patients who were uncooperative for examination.

## RESULTS

Seventy nine adults were diagnosed with leukemia at the Hematology/Immunology clinic of the University of Nigeria Teaching Hospital during the study period. Of these, seven patients comprising four males and three females failed to satisfy the inclusion criteria and were excluded from participation. The remaining 72 subjects with leukemia comprised 45 males and 27 females (male to female ratio =1.7:1). Mean age of the cohort was 32.7+ 9.8 years (range, 18 years to 72 years) constituted the study subjects or participants. The cohort demographics are presented in Table 1.

**Table 1:** Age and sex distribution of 72 leukemia patients

Age (years)	Sex		Total(%)
	Male	Female	
16 – 25	13	7	20 (27.8)
26 – 35	12	6	18 (25.0)
36 – 45	10	8	18 (25.0)
46 – 55	4	3	7 (9.7)
56 – 65	4	1	5 (6.9)
66 – 75	2	2	4 (5.6)
Total (%)	45 (62.5)	27 (37.5)	72 (100.0)

There were 42 new and 30 existing leukemic patients. The distribution of subgroups of leukemia was: CML in 28 (38.8%) subjects, CLL in 20 (27.8%) subjects, ALL in 13 (18.1%) subjects, AML in nine (12.5%) subjects and BPL in two (2.8%) subjects.

Sixty four (88.9%) subjects were undergoing cytotoxic drug therapy while eight (11.1%) subjects were not undergoing any form of treatment prior to ophthalmic evaluation. None of the study subjects underwent TBI or BMT therapy.

Leukemia-related ophthalmic lesion, in one or both eyes, was present in 56 (77.8%) subjects consisting of 44 new and 14 old patients, giving a 77.8% prevalence of leukemic ophthalmopathy. The remaining 16 (22.2%) subjects had no clinical evidence of leukemic ophthalmopathy.

Some subjects had more than one manifestation in one or both eyes. The ophthalmic manifestations of leukemia tended to involve more of the posterior than the anterior segment structures of the eye, and resulted more from secondary hematologic complications rather than primary leukemic infiltration [Table 2]. None of the subjects had orbital or neuro-ophthalmic manifestation of leukemia. Leukemic ophthalmic involvement was symptomatic in 38 (66.9%) and asymptomatic in 18 (32.1%) of subjects.

**Table 2:** Leukemia-related ophthalmologic manifestations in 72 leukemia patients

Ocular structure involved	Manifestation	No (%) of patients (n=72)
Anterior segment		
Conjunctiva	Pallor	20(27.8)
	Sub-conjunctival hemorrhage	14(19.4)
	Vascular dilatation/tortousity	2(2.8)]
Posterior segment		
Vitreous	Synchisis scintillans	3(4.2)
	Vitreous hemorrhage	5(6.9)
Optic disc	Pallor	5(6.9)
	Edema	2(2.8)
	Hemorrhage	2(2.8)
Retina		
Vessels	Vascular dilatation/tortousity	36(50.0)
Macular	Hemorrhage	5(6.9)
	Edema	2(2.8)
Background	Intra-retinal hemorrhage	12(16.7)
	Hard exudates	4(5.6)
	Soft exudates(cotton wool spots)	5(6.9)
	Leukemic infiltrates	2(2.8)
	Refractile spots	2(2.8)
	Edema	1(1.4)

The 56(77.8%) {males = 30(53.6%); females = 26(46.4%)} leukemia subjects who had ophthalmic manifestation of leukemia consisted of 22 cases of CML, 17 of CLL, 11 of ALL, five of AML, and one of BPL.

The prevalence of leukemic ophthalmopathy by type of leukemia was CML in 22/28 (78.6%) subjects, CLL in 17/20 (85.0%) subjects, ALL in 11/13 (84.6%) subjects, AML in 5/9(55.6%) subjects, and BPL in 1/2(50.0%) subjects. The prevalence of leukemic ophthalmopathy was higher in subjects with chronic (39/48, 81.3% subjects) leukemia than acute (16/22, 72.7% subjects) leukemia. Of the 56 subjects with leukemic ophthalmopathy, 38 (67.9%) had normal vision, 12(21.4%) were visually impaired, while 6 (10.7%) had acuity blindness. This translates to 32.1% (18/56) prevalence of visual loss.

Optic atrophy (*n*=7), macular hemorrhage (*n*=6), macular edema (*n*=3), and vitreous hemorrhage (*n*=2) accounted for leukemia-related vision loss.

Intraocular pressure ranged from 15 to 20 mmHg (normal range, 10 – 21 mmHg) in all the subjects. None (0.0%) of the participants had any abnormality of the pre-corneal tear film.

Miscellaneous ocular comorbidities not related to leukemia were seen in 34/72 (47.2%) subjects. These comprised arcus senilis (n =15), pterygium (n =10), cataract (n = 5) and age- related macular degeneration (n = 4). They caused vision loss in three subjects: one case of cataract and two cases of atrophic age-related macular degeneration.

The hospital prevalence of chronic leukemia was significantly higher than that of acute leukemia (66.7% vs 33.3%, *P* < 0.05) and leukemic ophthalmopathy was significantly associated with chronic rather than acute leukemia (70.9% *vs* 29.1%, *P* <0.05). There was no significant inter-sex difference in the prevalence of leukemia (males vs females: 62.5% vs 37.5%, *P* = 0.0822) and tendency to develop leukemic ophthalmopathy (53.6% vs 46.7%, *P* = 0.6624). The ophthalmic manifestations of leukemia more frequently involved the posterior than the anterior segment of the eye (70.5% *vs* 29.5% respectively, *P* <0.05) and were statistically significantly more often resulting from secondary hematologic complications than primary ocular infiltration (98.4% *vs* 1.6% respectively; *P* < 0.05).

## DISCUSSION

The demographic characteristics of our study cohort showed a preponderance of males; however, there were no statistically significant gender-based differences in the prevalences of leukemia and leukemic ophthalmopathy. This demographic trend is similar to reports in Ethiopia^12^ and Malaysia^11^ but could not be compared with the findings from a similar prospective study in USA^3^ which failed to report on their cohorts’ demographic profile. The observed sex distribution could be due to natural sex prevalence pattern of leukemia or the established pro-male gender inequity in access to health care^13^ or both. This seems to suggest the need to identify and overcome gender-related healthcare access barriers, and has important implications for health care planners and implementers.

Consistent with a previous report,^14^ and in keeping with the natural prevalence pattern of leukemia,^1^ more cases of chronic than acute leukemia were seen in our study. Additionally, acute leukemia often run a rapidly fatal course compared with chronic leukemia which is further aggravated in the resource-deficient African setting by pre-presentation care-pathway delays with attendant adverse implications for prognosis.^1^,^4^,^15^ These factors might further explain the observed prevalence pattern in our study.

The 77.8% prevalence of leukemic ophthalmopathy documented in the present study is comparable to 69.0% reported by Alemayehu *et al*,^12^ but differed markedly from 35.4% reported by Reddy *et al*,^11^ and 39.0% in a series by Schachat *et al*.^16^ The observed disparity could be attributed to the difference in case mix between the present study and those of Reddy *et al*, and Schachat *et al*. While both studies included children who are known to have a comparatively higher prevalence of acute leukemia with its associated higher incidence of leukemic ophthalmopathy,^2^ the present report is on adult leukemia patients only. The paradoxical observation in the present report, and that reported by Alemayehu *et al*,^12^ also in Africa, could be due to inherently higher prevalence of leukemic ophthalmopathy among black Africans or the consequence of deficient human and material resources needed for timely and effective management of systemic leukemia in Africa.^17^,^18^

In the present study, the spectrum of leukemic ophthalmopathy showed a preponderance of posterior over anterior segment manifestations. These resulted predominantly from secondary hematologic complications caused either by the systemic leukemia or its treatment rather than primary leukemic ocular infiltration.^16^,^17^ Orbital and adnexal manifestations were not documented in the present study and neuro-ophthalmic lesions were rare. Retinal vascular dilation/tortousity and retinal hemorrhage were the leading posterior segment lesions while conjunctival pallor and sub-conjunctival hemorrhage were the common anterior segment signs seen [Table 2]. These findings corroborate the spectrum of leukemic ocular involvement reported elsewhere in America,^2^,^16^ Asia,^11^ and Africa^12^ but differed from the high rate of primary neoplastic leukemic ocular infiltration in an autopsy series reported in America.^10^

Similar to previous ante-mortem studies,^2^,^11^,^12^,^16^ the present report documented only the clinically evident leukemia-related ocular lesions thus probably bypassing the occult manifestations. On the other hand, previous postmortem studies entailed a histological examination of the choroid, the most common site of infective or neoplastic leukemic ocular infiltration.^2^,^16^ This has implications for ophthalmologists performing ophthalmic evaluation of leukemia patients and seems to suggest that mandatory periodic eye examination is necessary, despite apparent non-involvement of eye. Furthermore, to enhance the clinician’s diagnostic capability to detect occult subclinical lesions, the authors suggest inclusion of ocular B-scan ultrasonography, computed tomography (CT) scan, and magnetic resonance imaging (MRI) in the routine screening protocol for leukemic ophthalmopathy.

The prevalence of miscellaneous ocular co- morbidities unrelated to leukemia was 47.2% and these were more frequently located in the anterior segment of the eye. This is comparable with 36.0% reported in an Ethiopian study^12^ but differs from the 21.0% documented by Schachat *et al*,^16^ while evaluating the eyes of leukemic adults and children in USA. The cohort demographics of the present study are similar to the Ethiopian survey but differed from that of Schachat *et al*,^16^ as described above. The exclusion of children from the present study probably explains the discrepant observations between the present study and Schachat *et al*’s^16^ study since majority of the co-morbid diseases encountered are age-related. This result suggests that ophthalmic evaluation in the setting of leukemia should go beyond routine clinical search for evidence of leukemic ophthalmopathy.

Overall, the prevalence of vision loss in patients with leukemic ophthalmopathy was 37.5%. This was caused by leukemic ocular involvement in 32.1%, and miscellaneous ocular co-morbidities in 5.4%. This differed from the 5.0% prevalence of overall vision loss observed by Schachat *et al*.^16^ The difference in cohort demographics coupled with differential access to standard care, which obviously favored the subjects in Schachat *et al*’s^16^ study, could explain the observed disparity in prevalence of vision loss. The contributions of leukemic ophthalmopathy and miscellaneous ocular co-morbidities to vision loss in the two studies could not be compared since the report by Schachat *et al*^16^ did not document the contribution of ocular co-morbidity to vision loss. The comparatively high prevalence of vision loss in the present study further underscores the need for periodic comprehensive ophthalmic examinations, and prompt treatment of blinding and vision threatening eye conditions, in leukemia patients.

The hospital-based nature of this study, its comparatively small sample size despite sufficiently long study period, and non-utilization of ocular B-scan ultrasound, CT or MRI during ophthalmic evaluation present limitations to the conclusions drawn from this work.

The prevalence of leukemic ophthalmopathy at UNTH, Enugu, is high. Ophthalmic leukemic involvement is more common in males, chronic leukemia, and tends to affect more of ocular posterior segment structures. Orbital, neuro-ophthalmic, and adnexal leukemic manifestations are rare. Miscellaneous ocular co-morbidities are frequent. Leukemic ophthalmopathy and co-morbid ocular disease contribute significantly to vision loss. These findings have clinical implications for all health care providers involved in the management of leukemia. Prompt initial and regular periodic radiology-assisted comprehensive ophthalmic evaluation is recommended in all leukemia patients.

## References

[1] Catovsky D, Weatheral DJ, Ledingham JG, Warell DA (1996). Chronic lymphocytic leukemias and other leukemias of mature B and T Cells. Oxford Textbook of Medicine.

[2] Gordon KB, Rugo HS, Duncan JL, Irvine AR, Howes EL, O’Brein JM (2001). Ocular Manifestations of leukemia: Leukemic infiltration versus infectious process. Ophthalmology.

[3] Karesh JW, Goldman EJ, Reck K, Kelman SE, Lee EJ, Schiffer CA (1989). A prospective ophthalmic evaluation of patients with acute myeloid leukemia: Correlation of ocular and hematologic findings. J Clin Oncol.

[4] Sharma T, Grewal J, Gupta S, Murray PI (2004). Ophthalmic manifestations of acute leukemias: The Ophthalmologist’s role. Eye.

[5] Margulies LJ (1994). Ocular manifestations of Cardiovascular and hematologic disorders. Curr Opin Ophthalmol.

[6] Charif-Chefchaouni M, Belmeki M, Hajji Z, Tahiri H, Amrani R, Bakkali M (2002). Ophthalmic manifestations of acute leukemia. J Fr Ophtalmol.

[7] Claes K, Kestelyn P (2000). Ocular manifestations of graft versus host disease following bone marrow transplantation. Bull Soc Belge Ophtalmol.

[8] Kiura K, Niiya K, Harada M (2002). Ocular manifestations of acute graft-versus-host disease after allogeneic peripheral blood stem cell transplantation. Int J Hematol.

[9] Decker EP, Burnstine RA (1993). Leukemic relapse presenting as acute unilateral hypopyon in acute lymphocytic leukemia. Ann Ophthalmol.

[10] Leonardy NJ, Rupani M, Dent G, Klintworth GK (1990). Analysis of 135 autopsy eyes for ocular involvement in leukemia. Am J Ophthalmol.

[11] Reddy SC, Jackson N, Menon BS (2003). Ocular involvement in Leukemia: A study of 288 cases. Ophthalmologica.

[12] Alemayehu W, Shamebo M, Bedri A, Mengistu Z (1996). Ocular manifestations of leukemia in Ethiopians. Ethiop Med J.

[13] Adamson J, Ben-Shlomo Y, Chaturvedi N, Donovan J (2003). Ethnicity, socioeconomic position and gender- do they affect reported health-care seeking behavior?. Soc Sci Med.

[14] Sing AD (2003). The prevalence of ocular disease in chronic lymphocytic Leukemia. Eye.

[15] Lee SS, Robinson MR, Morris JC, Mirtschung BC, Shen D, Chan C (2004). Conjunctival involvement with T-cell prolymphocytic leukemia: Report of a case and Review of the Literature. Surv Ophthalmol.

[16] Schachat AP, Markowitz JA, Guyer DR, Burke PJ, Karpe JA, Graham ML (1989). Ophthalmic manifestations of Leukemia. Arch Ophthalmol.

[17] Kincaid MC, Green WR (1983). Ocular and Orbital involvement in leukemia. Surv Ophthalmol.

[18] Rosenthal AR (1983). Ocular manifestations of Leukemia: A Review. Ophthalmology.

